# Supplementary material for: In vivo imaging of the barrier properties of the glia limitans during health and neuroinflammation
Source: Nat Commun. 2025 Oct 7;16:8895. doi: 10.1038/s41467-025-63945-7 (PMC12504616; doi:10.1038/s41467-025-63945-7)
Supplement: Supplementary file 1 — Supplementary Information [file 41467_2025_63945_MOESM1_ESM.pdf]

Supplementary File

In vivo imaging of the barrier properties of the glia limitans during health and neuroinflammation

Pauline Hélie-Legoupil[1,3,#], Florencia Kloster[1,#], Javier Pareja[1], Mykhailo Vladymyrov[1], Josephine A. Mapunda[1,4,5], Elisa Bouillet[1], Yannik Oetiker[1], Irene Spera[1,6], Sara Barcos[1], Amandine Brenna[1], Adolfo Odriozola[2], Alyssa Baert[1,7], Christoph Fankhauser[1], Beat Haenni[2], Steven T. Proulx[1], Benoît Zuber[2], Urban Deutsch[1] and Britta Engelhardt[1,*]

[1]Theodor Kocher Institute, University of Bern, Bern, Switzerland, [2]Institute of Anatomy, University of Bern, Bern, Switzerland

[3]present address: Vetsuisse Faculty, Department of Veterinary Anatomy, University of Bern, Switzerland; [4,5]present addresses: [4]School of Life Sciences and Bioengineering, Nelson Mandela African Institution of Science and Technology, Arusha, Tanzania and [5]The Hormel Institute, University of Minnesota, Austin MN, USA; [6]present address: Department of Biomedicine, Ocular Pharmacology and Physiology, University of Basel, Switzerland ; [7]present address: Department for Neuroimmunology and Multiple Sclerosis Research, University Göttingen, Germany

[#]: equal contribution
*: corresponding author

Correspondence:
Prof. Dr. Britta Engelhardt
Theodor Kocher Institute, University of Bern, Bern, Switzerland
E-Mail: britta.engelhardt@unibe.ch

Supplementary Figures

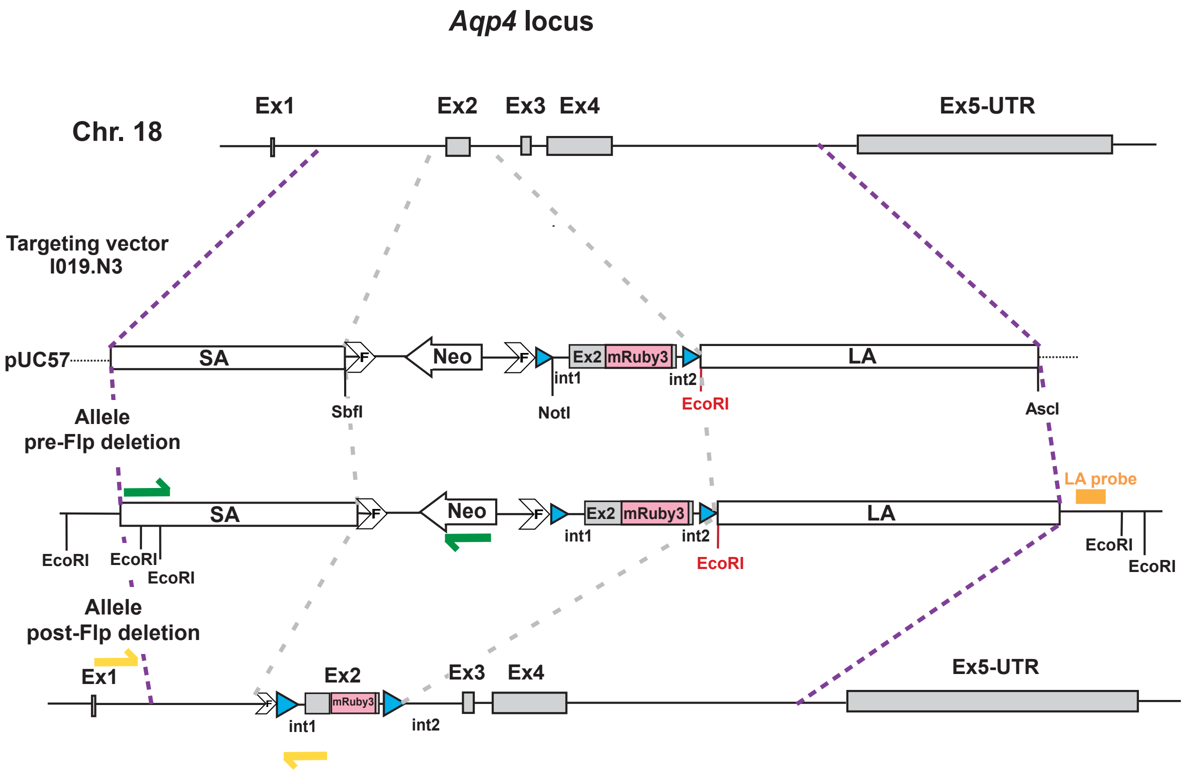

Supplementary Figure 1: Gene targeting strategy for the Aqp4-mRuby3 knock-in fluorescent reporter mice.

At the top of the figure the genomic structure of the mouse *Aqp4* locus on chromosome (Chr.) 18 is shown, with each exon symbolized by a grey box. Below that, the targeting vector is depicted. The AscI recognition site indicated was used for linearization prior to electroporation into embryonic stem (ES) cells. To facilitate the incorporation of the mRuby3 insert into exon 2 by homologous recombination, the vector contained a short arm (SA) of 2.5 kB and a long arm of homology (LA) of 4.8 kB., represented by white boxes. In between the SA and the LA was a fragment consisting of a FRT flanked (F, white arrowheads) neomycin resistance cassette (Neo), an upstream loxP site (turquoise triangle) followed by a short segment from intron 1 and the 5’ portion of exon 2 containing an mRuby3 open reading frame flanked by sequences coding for flexible linkers (3xGGGS; G = Gly; S = Ser) and a downstream lox2272 site (turquoise triangle). The bottom-middle part of the figure shows the correctly targeted allele. Positions of primers (green) used for PCR screening of candidate targeted ES cell clones and the position of the probe (orange) used in Southern Blotting of EcoRI digested genomic DNA. EcoRI restriction sites are indicated, the EcoRI site present only in the targeted allele is indicated in red. The allele resulting from deletion of the neomycin selection cassette following breeding with the Flp-deleter mouse strain is shown in the bottom panel. Screening by PCR was performed to confirm deletion of the Neo cassette (primers in yellow). SA = short arm, LA = long arm, F = FRT sites, Ex = exon, Neo = neomycin, UTR = untranslated region, int = intron, Chr = chromosome.

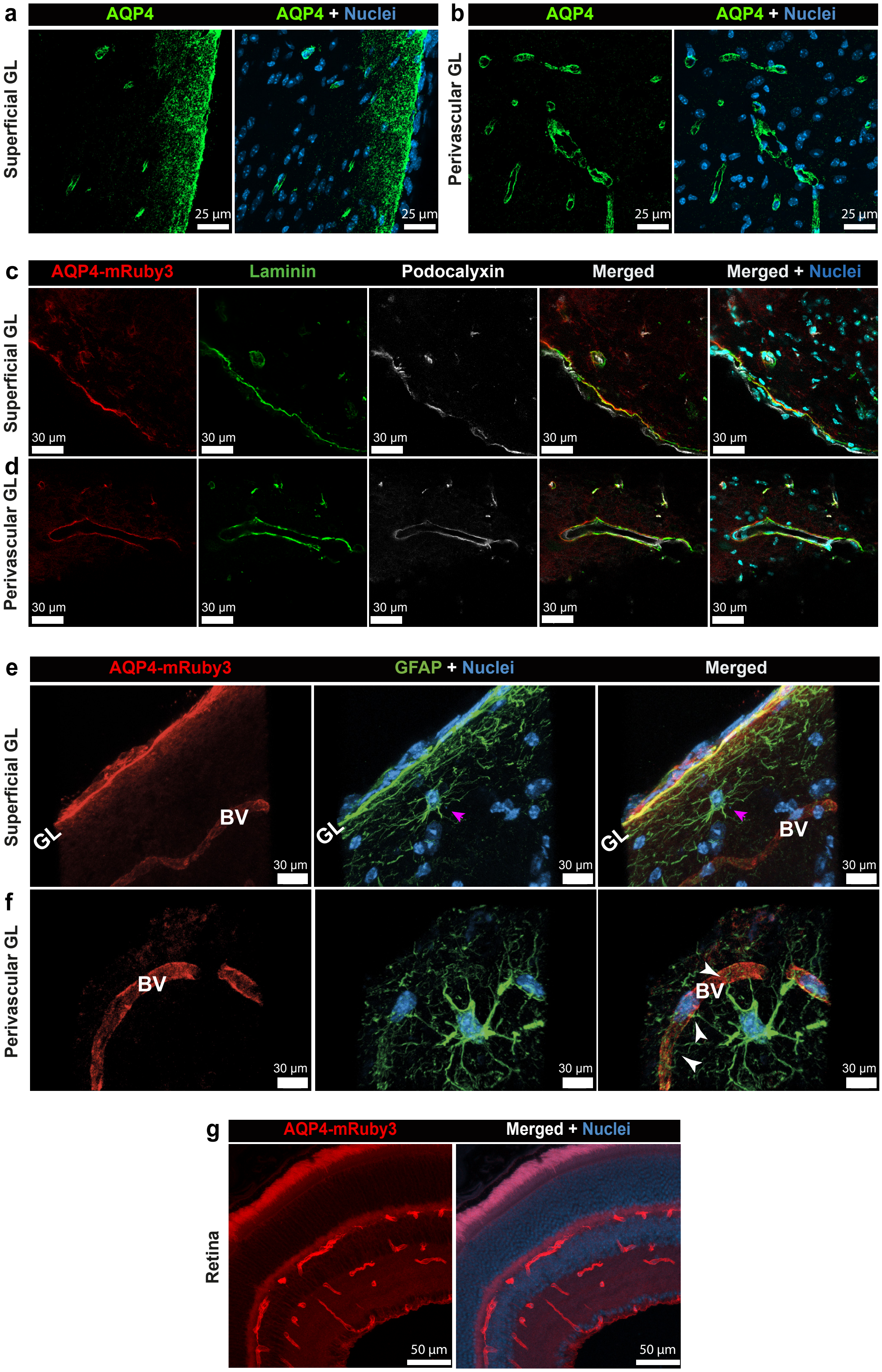
a
AQP4
AQP4 + Nuclei
Superficial GL
25 µm
b
AQP4
AQP4 + Nuclei
Perivascular GL
25 µm
c
AQP4-mRuby3
Laminin
Podocalyxin
Merged
Merged + Nuclei
Superficial GL
30 µm
d
Perivascular GL
30 µm
e
AQP4-mRuby3
GFAP + Nuclei
Merged
Superficial GL
GL
BV
30 µm
f
Perivascular GL
BV
30 µm
g
AQP4-mRuby3
Merged + Nuclei
Retina
50 µm

Supplementary Figure 2: AQP4-mRuby3 localized to the superficial and perivascular glia limitans.
a-b: Immunofluorescence staining for AQP4 (green) of 20 µm thick brain cryosections from C57BL/6J mice. Nuclei (DAPI) are shown in blue. MIP images from the surface (**a**) and cortex (**b**) showing the superficial and perivascular glia limitans. Data is representative of three mice. **c-f:** MIP of confocal images of immunofluorescence staining of 20 µm thick brain cryosections collected from healthy Aqp4-mRuby3 mice. The AQP4-mRuby3 signal of the glia limitans (GL) is visualized in red and nuclei were stained with DAPI (blue). **c-d:** Immunofluorescence staining for podocalyxin (white) and laminin (green) of brain cryosections from Aqp4-mRuby3 mice. Colocalization is observed for the AQP4-mRuby3 and the laminin signals at the basement membrane at the superficial GL (**c**) and at the level of the individual vessels in the perivascular GL (**d**). Data is representative of two mice. **e-f:** Anti -glial fibrillary acidic protein (GFAP) staining detects astrocytes (green). The endogenous AQP4-mRuby3 signal is detected on $GFAP^{+}$ astrocytes (pink arrowhead) at the surface of the brain (**e**). The astrocyte endfeet form the perivascular GL (white arrowheads) surrounding penetrating vessels (**f**). Data is representative of three mice. **g:** Representative image of the mouse retina from 20 µm thick decalcified head cryosections from female Aqp4-mRuby3 mice suffering from aEAE (peak, score=1), data representative of 1 mouse. Endogenous signal from AQP4-mRuby3 protein is shown in red. Nuclei (DAPI) are shown in blue. GL = glia limitans, BV = blood vessel.

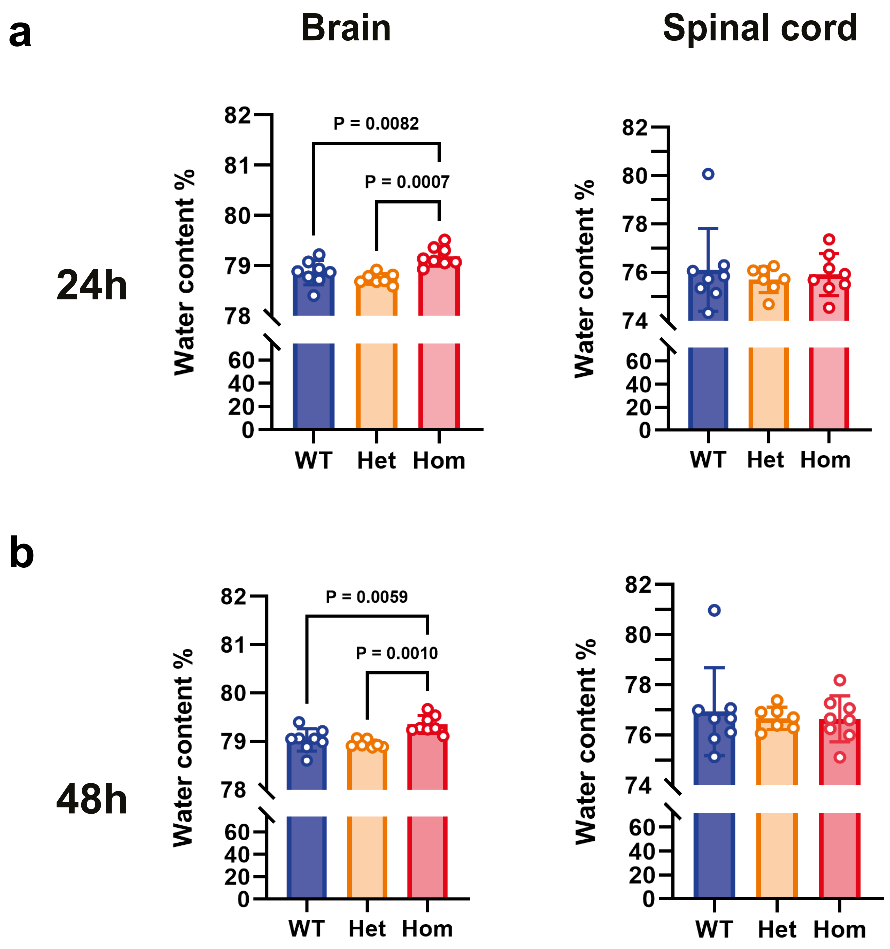

Supplementary Figure 3: CNS water content in Aqp4-mRuby3 reporter mice.

Water content in the brains and spinal cords of heterozygous (Het) and homozygous (Hom) Aqp4-mRuby3 reporter mice and wild-type C57BL/6 littermates (WT) was evaluated. Bar graphs show water content (in %) of brains and spinal cords of female WT (n=8), Het (n=7) and Hom (n=8) reporter mice 24h post-drying time (**a**) and of brains and spinal cords of female WT (n=7), Het (n=7) and Hom (n=8) reporter mice 48h post-drying time (**b**), respectively. Water content was calculated as described in Methods. Data are shown as mean ± SD, individual symbols represent one mouse. Data were analyzed using a one-way ANOVA with Tukey's multiple comparisons test. Source data including exact p values are provided as a Source Data file.

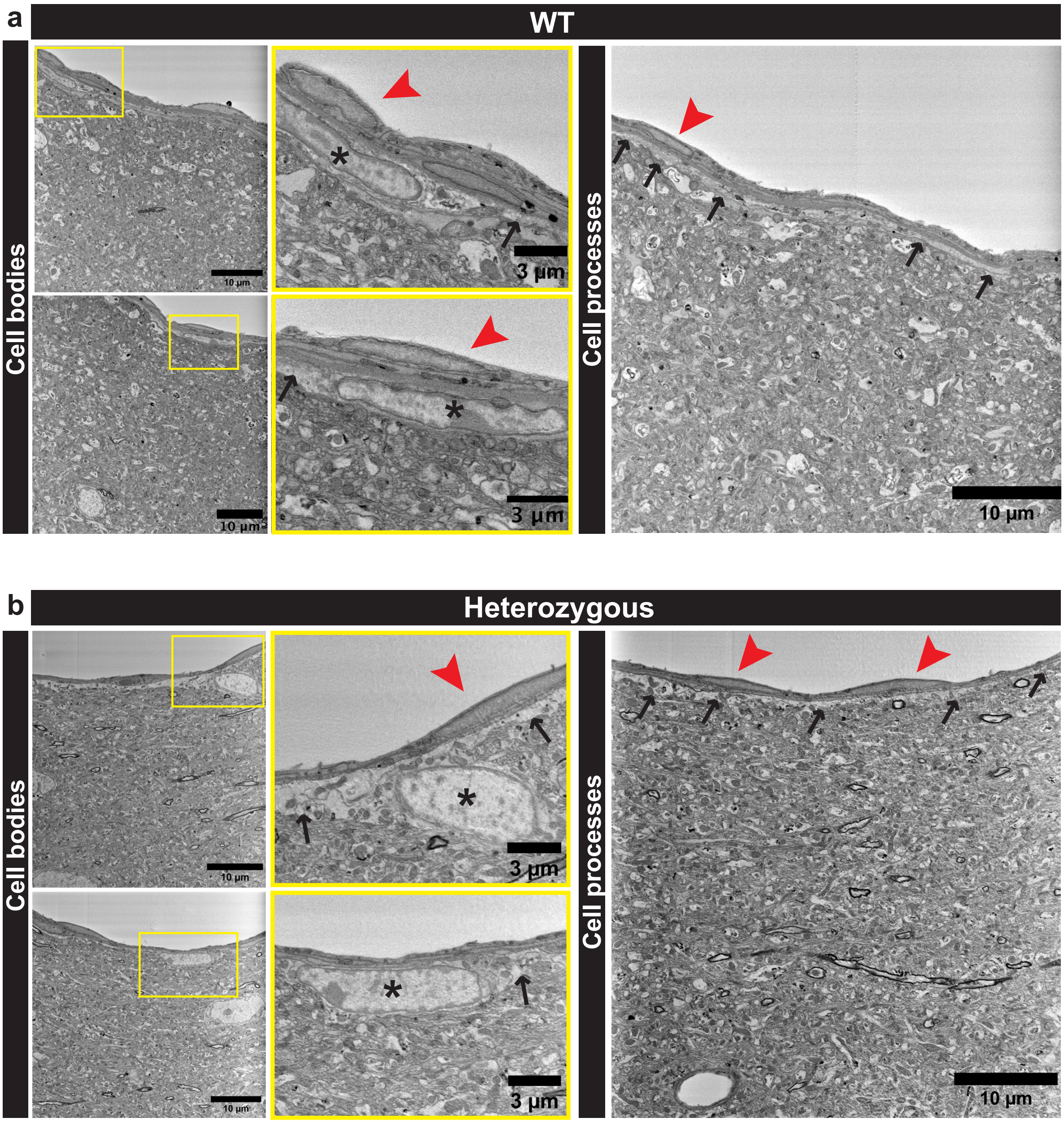

Supplementary Figure 4: Astrocyte cell bodies extending cellular processes form the superficial glia limitans.

Serial block face scanning electron microscopy (SBF-SEM) analysis of the superficial glia limitans in WT (**a**) and heterozygous Aqp4-mRuby3 mice (**b**) is shown (n=1). For each genotype, two distinct representative areas are shown. In the left panel, images depicting astrocyte cell bodies (asterisks) positioned directly below pial fibroblasts (red arrowheads) are shown. Localization of the zoom-ins is highlighted by yellow rectangles focusing on the respective astrocyte nucleus (asterisks). Right panel illustrates regions where the superficial glia limitans is exclusively formed by astrocyte cell processes (black arrows).

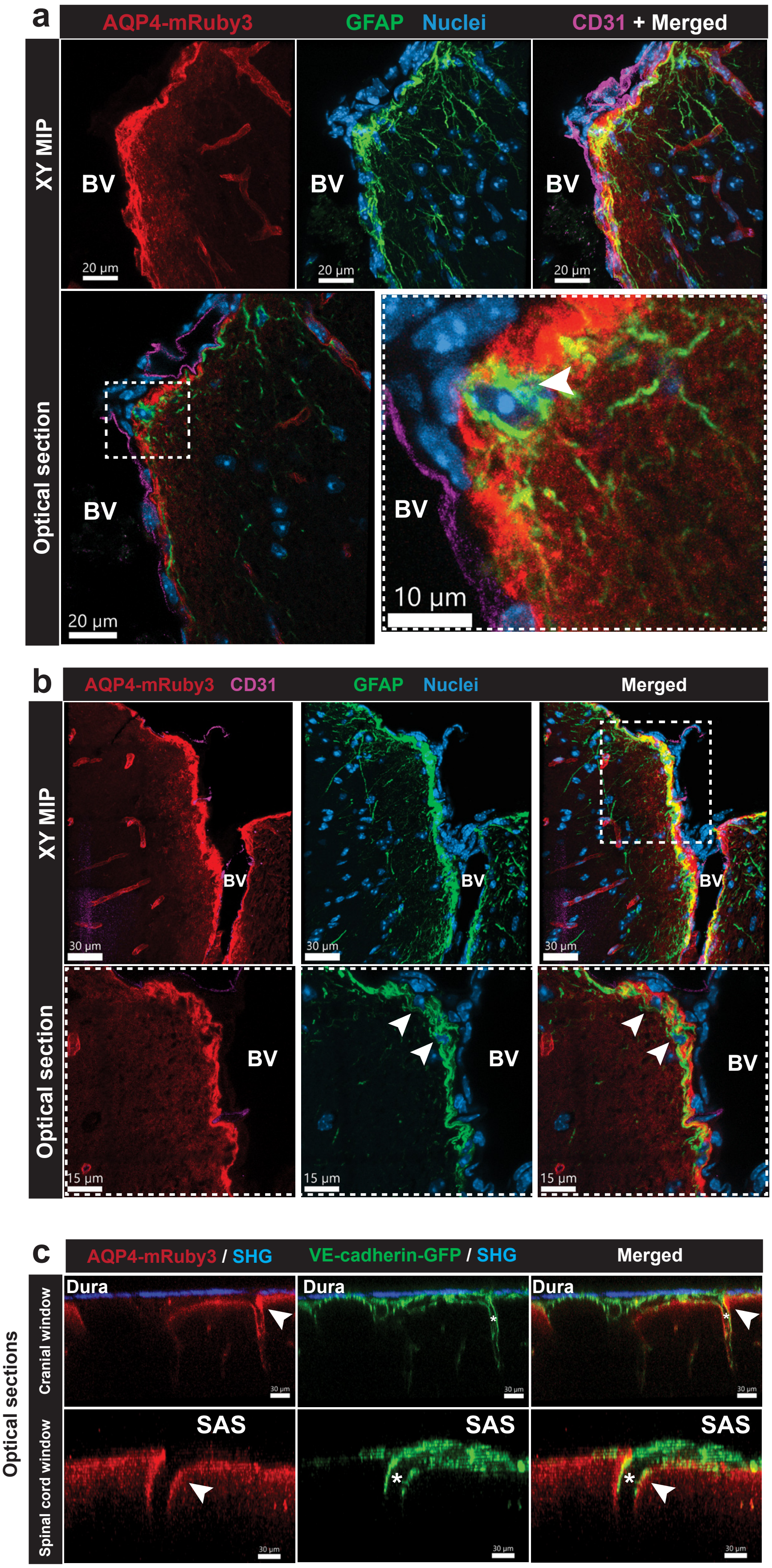
a
AQP4-mRuby3
GFAP Nuclei
CD31 + Merged
XY MIP
Optical section
BV
20 µm
10 µm
b
AQP4-mRuby3 CD31
GFAP Nuclei
Merged
XY MIP
Optical section
BV
30 µm
15 µm
c
AQP4-mRuby3 / SHG
VE-cadherin-GFP / SHG
Merged
Optical sections
Cranial window
Spinal cord window
Dura
SAS
30 µm

Supplementary Figure 5: Polarized localization of the AQP4-mRuby3 fusion protein in astrocytes at the transition zone between superficial and perivascular glia limitans in healthy heterozygous Aqp4-mRuby3 reporter mice.

a-b: Immunofluorescence staining for GFAP (green) as marker for astrocytes and endothelial CD31 (magenta) in 40 µm thick decalcified coronal skull cryosections from heterozygous Aqp4-mRuby3 reporter mice imaged by confocal microscopy. The AQP4-mRuby3 signal is visible in red, astrocytes in green, endothelial cells in magenta and nuclei (DAPI) are shown in blue. Top panel: XY MIP images showing the glia limitans transition that occurs when a leptomeningeal blood vessel (BV) enters the brain parenchyma. Bottom panel: Optical sections allow detection of astrocyte cell bodies at the superficial glia limitans (white arrowheads) and zoom-ins (dotted squares) enable visualization of polarized localization of AQP4-mRuby3 in the astrocytes towards the outer surface. Data is representative of 3 mice. **c:** Transition area of superficial to perivascular glia limitans in CNS border reporter mice (Aqp4-mRuby3; VE-cadherin-GFP) imaged by 2P-IVM in cranial (top panel) and spinal cord window (bottom panel) preparations. Overlay of images with excitation wavelengths of 920 nm and 1045 nm, respectively. YZ MIPs illustrating the transition of penetrating vessels (asterisks) as they reach the parenchyma from the surface, with the transition from superficial to perivascular glia limitans (white arrowhead) marked by the AQP4-mRuby3 signal (red). The VE-cadherin-GFP signal (green) depicts the adherens junctions from the endothelial cells forming blood vessels and from the leptomeningeal fibroblasts. Data are representative of 3 mice per preparation. BV = blood vessel, SAS = subarachnoid space, SHG = second harmonic generation, MIP = maximum intensity projection.

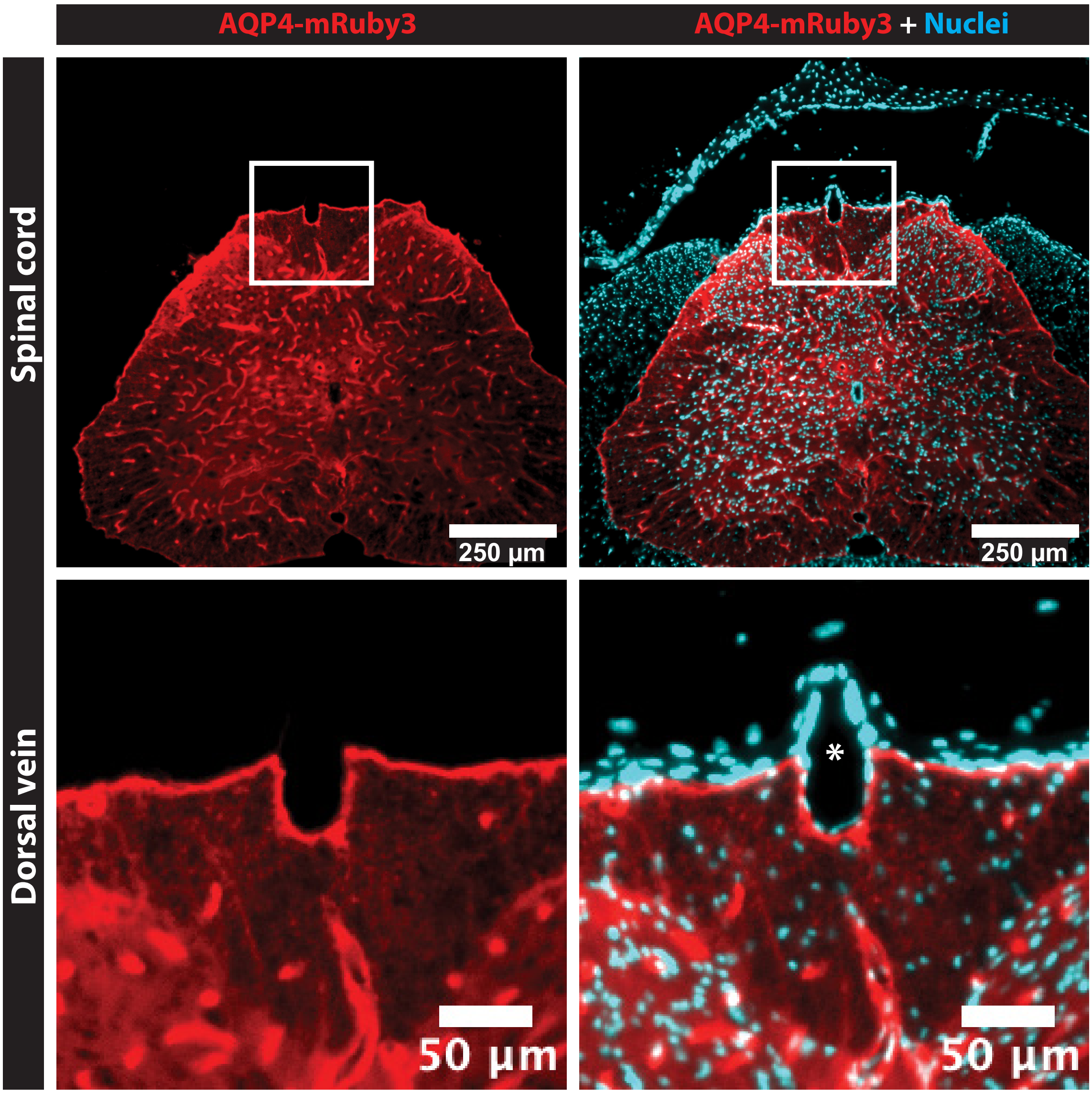

Supplementary Figure 6: AQP4-mRuby3 expression is continuous over the surface of the spinal cord. Epifluorescence imaging of decalcified sections of the vertebral column of a heterozygous Aqp4-mRuby3 knock-in mouse. Images from **Fig. 1d** show the dorsal vein in more detail. The endogenous AQP4-mRuby3 signal is visible in red and DAPI staining highlights nuclei in blue. The boxed area in the upper panels is shown in higher magnification below. Asterisk marks the lumen of the dorsal spinal cord vein. Data is representative of 3 mice.

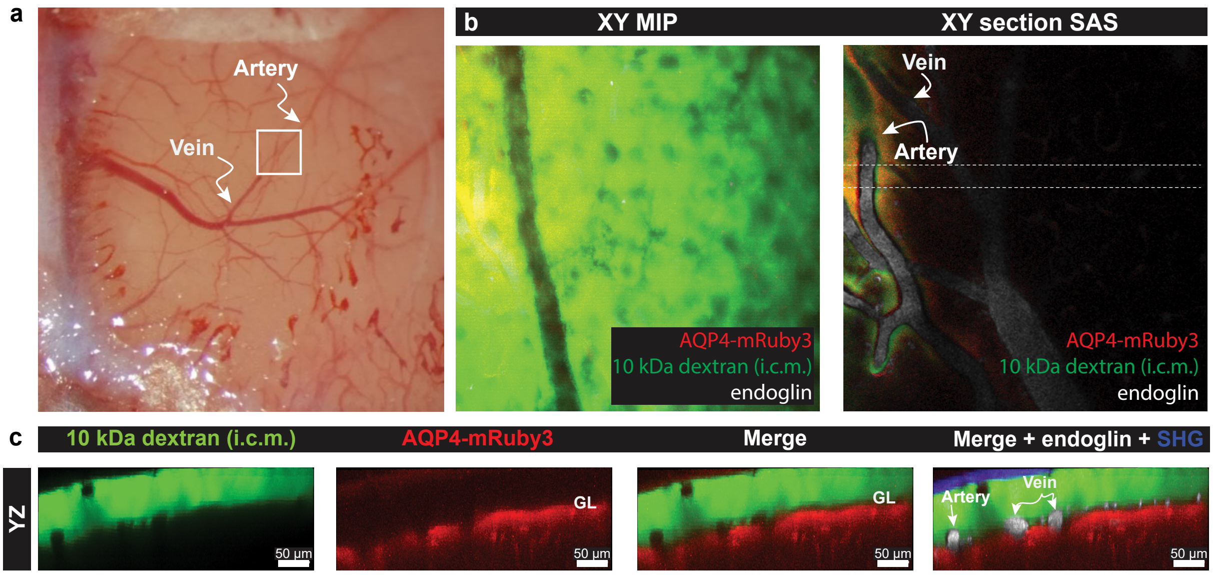

Supplementary Figure 7: 2P-IVM of CSF infused tracers in Aqp4-mRuby3 mice through skull thinning. Aqp4-mRuby3 mice received a cisterna magna injection of a 10 kDa FITC-dextran (green) and an anti-endoglin-AF633 antibody (white) via a carotid artery catheter to label the luminal wall of the vasculature. 2P-IVM of the brain surface through a thinned skull was performed. **a**: Representative image of the thinned skull. Veins were identified as vessels branching into the superior sagittal sinus with a blood flow towards the sinus. Arteries were identified as vessels branching from the lateral side of the brain with a blood flow towards smaller caliber vessels. White box shows the field of view imaged in (**b**) under the 2P microscope. **b-c:** 2P images of the white box in (**a**) acquired with 2P excitation wavelengths of 860 and 980 nm at 20 minutes after cisterna magna injection. **b**: Left panel shows the XY MIP of the field of view in the white box (**a**). Right panel shows a 2 µm thick optical section from the left panel. AQP4-mRuby3 depicting the glia limitans is visible in red. **c**: YZ MIP from the area between the dashed lines in (**b**). SHG is shown in blue. MIP = maximum intensity projection, SAS = subarachnoid space, i.c.m = intra-cisterna magna, SHG = second harmonic generation. Data is representative of 2 independent experiments.

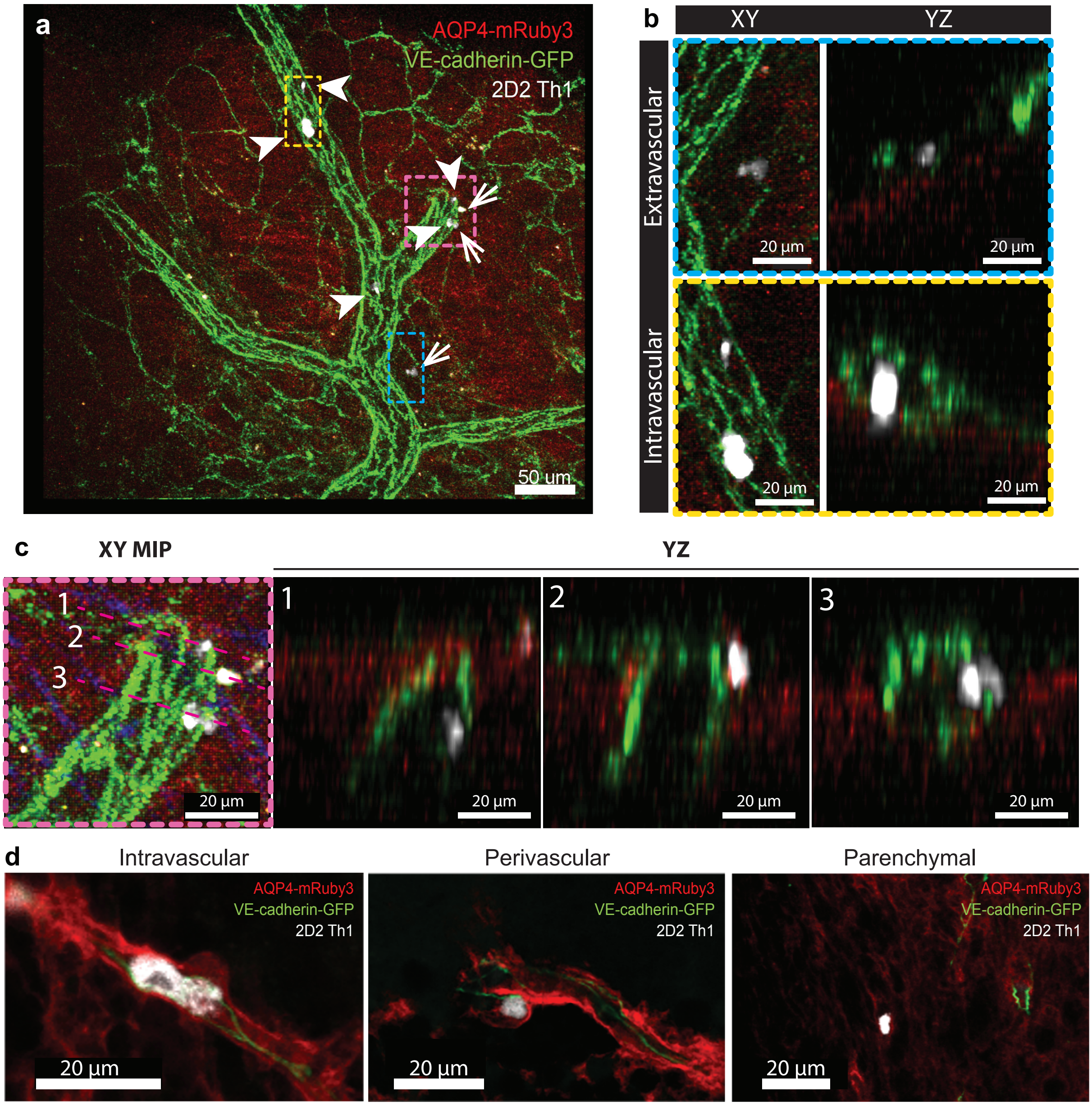

Supplementary Figure 8: CNS border reporter mice allow distinction between CNS border patrolling from CNS infiltrating CD4 T cells during EAE.

EAE was induced in female CNS border reporter mice and 2P-IVM of the cervical spinal cord was performed at EAE peak (18-19 days p.i.). During 2P-IVM, 5 x 10^6 Deep-Red Cell Tracker labeled 2D2 Th1 cells were systemically injected via a carotid artery catheter. Images were acquired using simultaneously 2P excitation wavelengths of 920 and 1045 nm. **a**: XY MIP image of the 2P-IVM imaging of the cervical spinal cord at the peak of aEAE (n=3). Glia limitans (red), endothelial and leptomeningeal adherens junctions visible in green. 2D2 Th1 cells are shown in white. White arrows point to extravascular 2D2 Th1 cells. White arrowheads point to 2D2 Th1 cells localized within blood vessels. **b**: XY and YZ MIP images of the light blue and yellow insets from (**a**), showing an extravascular and an intravascular 2D2 Th1 cell respectively. **c**: XY MIP of the magenta inset in (**a**) showing several intra and extravascular 2D2 Th1 cells. YZ images show 2µm thick YZ images from position 1, 2 and 3. **d**: Confocal imaging of 100 µm thick brain vibratome sections border reporter mice at the peak of EAE. AQP4-mRuby3 signal is shown in red. VE-

cadherin-GFP signal is shown in green. 2D2 Th1 cells located within the blood vessels, perivascular space or brain parenchyma are shown in white. Data are representative of 3 mice. MIP = maximum intensity projection.

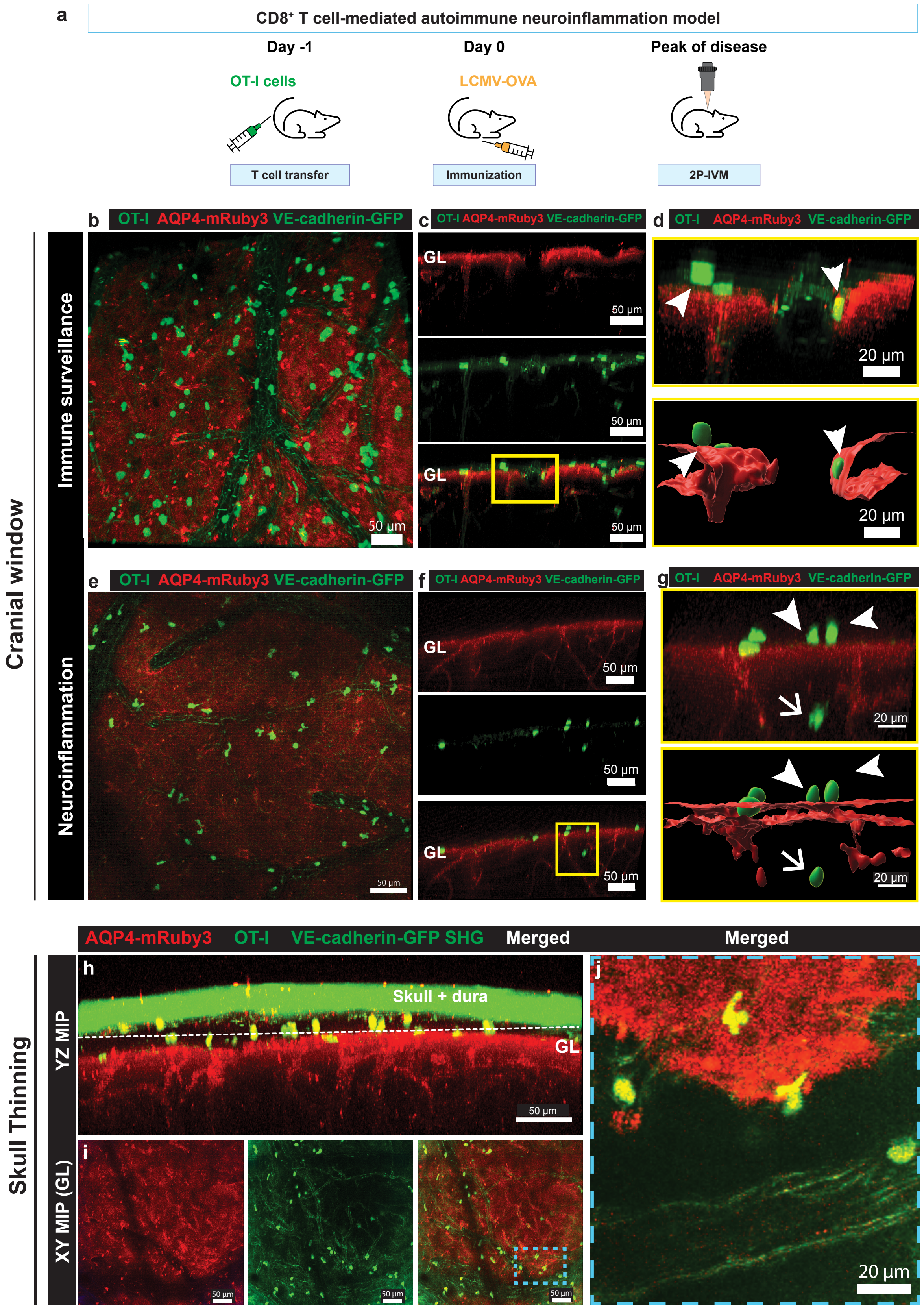

Supplementary Figure 9: 2P-IVM of CD8 T cells in brains of female CNS border reporter mice during immune surveillance and neuroinflammation.

a: Female CNS border reporter mice (**b-d**, **h-j**) or CNS border reporter; ODC-OVA mice (**e-g**) received an intravenous injection of 2 x 10^5 naïve GFP+ OT-I CD8+ cells (green) followed one day later by a peripheral

infection with LCMV-OVA. On day 7 after the LCMV-OVA infection, 2P-IVM of the brain surface through a cranial window (**b-g**) or thinned skull (**h-j**) was performed. The adherens junctions from endothelial cells, arachnoid mater and pia mater fibroblasts are visible in green due to their VE-cadherin-GFP signal. The glia limitans is visible in red due to the AQP4-mRuby3 signal. The remaining bone and dura mater are visible in green from to the SHG of the collagen type I fibers, due to the 2P excitation wavelength of 1045 nm (**h-j**). **b-g**: Representative XY MIP (**b, e**) YZ MIP (**c, d, f, g**) images of the brain surface are shown. White arrowheads point to CD8 T cells on top of the pia mater and glia limitans. White arrows point to parenchymal CD8 T cells below the glia limitans. **d, g**: Zoom-in of the yellow boxed areas in (**c, f**) showing the fluorescence signal (top panel) and the segmented surfaces (bottom panel) of the glia limitans and CD8 T cells. Surfaces were rendered with Imaris 9.8 software. Data were formed by overlaying images acquired with 2P excitation wavelengths of 920nm and 1045 nm. Data are representative of 4 mice per condition. **h-j**: Representative YZ MIP (**h**) and XY MIP (**i, j**) images are shown. XY MIPs are shown excluding the dura mater. **j**: 2µm thick optical section of the blue boxed inset from h. MIP = maximum intensity projection, SHG = second harmonic generation, GL = glia limitans. SAS = subarachnoid space, DV = dorsal vein.

Supplementary Table 1: information regarding the antibodies used for all experiments performed.

	Antibodies used for immunofluorescence stainings				
	**Antibody**	**Company**	**Clone number**	**Catalog number**	**Working concentration**
**Primary**	Rat anti-mouse CD45	Produced in house	M1/90	-	Undiluted hybridoma cell culture supernatant
	Rabbit anti-AQP4	Millipore	Polyclonal	AB2218	10 µg/mL
	Rabbit anti-mouse pan laminin	Novus	Polyclonal	NB300-144SS	5 µg/mL
	Rabbit anti-mouse laminin 1+2 antibody	Abcam	Polyclonal	ab7463	5 µg/mL
	Goat anti-mouse podocalyxin	R&D Systems	Polyclonal	AF1556	2 µg/mL
	Rabbit anti-glial fibrillary acidic protein (GFAP)	DAKO	Polyclonal	ZO334	24 µg/mL
	Goat IgG isotype	R&D Systems	Polyclonal	AB-108-C	3.3 µg/mL
	Normal rabbit IgG isotype	Invitrogen	Polyclonal	02-6102	10 µg/mL
	Rat IgG2a isotype	Pharmingen	R35-95	553927	10 µg/mL
	Rat anti mouse-CD31	Produced in house	Mec13.3	-	Undiluted hybridoma cell culture supernatant
**Secondary**	Goat anti-rabbit IgG (H+L) - Alexa Fluor 647	ThermoFisher Scientific	Polyclonal	A-21244	4 µg/mL
	Donkey anti-rabbit IgG - Cyanine5	Jackson ImmunoResearch	Polyclonal	711-175-152	7.5 µg/mL
	Donkey-anti goat IgG polyclonal - Alexa Fluor 647	Jackson ImmunoResearch	Polyclonal	705-605-003	7.5 µg/mL
	Donkey anti-rabbit IgG - Alexa Fluor 488	Invitrogen	Polyclonal	A32790	6.7 µg/mL
	Donkey anti-rat IgG - Alexa Fluor 647	Invitrogen	Polyclonal	A78947	6.7 µg/mL
	Donkey anti-rat IgG - Alexa Fluor 488	ThermoFisher	Polyclonal	A-21208	6.7 µg/mL
	Antibodies used for labelling of vascular lumen during 2photon-microscopy imaging				
	**Antibody**	**Company**	**Clone**	**Catalog number**	**Working concentration**
**Primary**	Rat-anti mouse endoglin - Alexa Fluor 633	produced and labelled in house	MJ7/18	-	40 µg/mouse
