# Supplementary material for: In vivo imaging of the barrier properties of the glia limitans during health and neuroinflammation
Source: Nat Commun. 2025 Oct 7;16:8895. doi: 10.1038/s41467-025-63945-7 (PMC12504616; doi:10.1038/s41467-025-63945-7)
Supplement: Supplementary file 2 — Description of Additional Supplementary Files [file 41467_2025_63945_MOESM2_ESM.docx]

**Description of additional supplementary files**

**Supplementary Movie 1: 2P-IVM of an Aqp4-mRuby3 mouse brain through a cranial window.**

Representative 3D reconstruction of the brain cortical surface from an Aqp4-mRuby3 mouse imaged with 2P-IVM through a cranial window after intravenous injection of 10 kDa FITC-dextran allowing to visualize the blood vessel lumen (green). Due to the acquisition settings with an excitation laser wavelength of 1045 nm, the SHG induced by the collagen type I fibers in the dura mater is visible in green. AQP4-mRuby3 is visible in red at the expected level of the glia limitans. This video is the source dataset of the images shown in Figure 4b. BV = blood vessel. Images are representative of a total of 4 mice.

**Supplementary Movie 2: 2P-IVM of an Aqp4-mRuby3 mouse brain through a skull thinning preparation.**

Representative 3D reconstruction of the brain cortical surface from an Aqp4-mRuby3 mouse imaged with 2P-IVM through a thinned skull after intravenous injection of 10 kDa FITC-dextran allowing to visualize the blood vessel lumen (green). Due to the acquisition settings with an excitation laser wavelength of 1045 nm, the SHG induced by the collagen type I fibers in the dura mater and remaining skull bone is visible in green. AQP4-mRuby3 is visible in red at the expected level of the glia limitans. Asterisks highlight the superficial glia limitans delineating the penetrating blood vessels. This video is the source dataset of the images shown in Figure 4d. BV = blood vessel. Images are representative of a total of 4 mice.

**Supplementary Movie 3: Serial block face scanning electron microscopy (SBF-SEM) imaging of superficial glia limitans of an Aqp4-mRuby3 mouse brain.**

Representative images of a z-stack acquired at 200 nm spacing intervals to visualize the superficial glia limitans ultrastructure from the brain of a heterozygous Aqp4-mRuby3 mouse imaged with SBF-SEM are shown. The superficial glia limitans exhibits a heterogeneous composition of areas mainly composed of astrocyte cell processes interspersed with astrocyte cell bodies located below pial fibroblasts. This video is the source dataset of the images shown in Supplementary Figure 4b**.** Scale bar: 10 µm.

**Supplementary Movie 4: 2P-IVM of an Aqp4-mRuby3 mouse cervical spinal cord.**

Representative 3D reconstruction of the cervical spinal cord surface from an Aqp4-mRuby3 mouse imaged with 2P-IVM after intravenous injection of a 10 kDa FITC-dextran allowing to visualize the blood vessel lumen (green). Due to the acquisition settings with an excitation laser wavelength of 1045 nm, the SHG induced by the collagen type I fibers in the dura mater, in the subpial space and the trabeculae are visible in green. AQP4-mRuby3 is visible in red at the expected level of the glia limitans. This video is the source dataset of the images shown in Figure 4f**.** SAS = subarachnoid space, DV = dorsal vein. Images are representative of a total of 5 mice.

**Supplementary Movie 5: 2P-IVM of the brain of a border reporter mouse through a cranial window.**

Representative 3D reconstruction of the brain cortical surface from a border reporter mouse (Aqp4-mRuby3; VE-cadherin-GFP) imaged with 2P-IVM through a cranial window. Due to the acquisition settings with an excitation laser wavelength of 1045 nm, the SHG induced by the collagen type I fibers in the dura mater is visible in green. AQP4-mRuby3 is visible in red at the expected level of the glia limitans. The VE-cadherin-GFP signal is visible in green at adherens junctions of the vascular endothelium and the arachnoid and pia mater fibroblasts. First, an XY maximal intensity projection (MIP) of the cranial window is shown from the top. Next a focus on a subarachnoid blood vessel (XY MIP), after digitally removing the dura and arachnoid (image of the same vessel) is shown. The vascular wall (green) and the glia limitans (red) are clearly distinguishable also on a YZ MIP (lateral view). Images are representative of a total of 3 mice.

**Supplementary Movie 6: 2P-IVM of the spinal cord of a border reporter mouse through a spinal cord window.**

Representative 3D reconstruction of the cervical spinal cord surface from a border reporter mouse (Aqp4-mRuby3; VE-cadherin-GFP) imaged by 2P-IVM through a spinal cord window. Due to the defined acquisition settings with simultaneous excitation laser wavelengths of 1045 nm and 900 nm the SHG from the dura mater collagen type I fibers is visible in green and blue on the surface. AQP4-mRuby3 is visible in red at the expected level of the glia limitans. The VE-cadherin-GFP signal is visible in green at the adherens junctions of the vascular endothelial cells and the arachnoid and pia mater fibroblasts. First, an XY MIP of the window is shown from the top and then after a rotation a YZ MIP (lateral view) is shown. After a description of each area and following the digital removal of the dura and arachnoid, the FOV from the movie focuses on a subarachnoid vessel (XY MIP). The vascular wall (green) and the glia limitans (red) are clearly distinguishable (also on a YZ MIP). SAS = subarachnoid space, BV = blood vessel. Images are representative of a total of 4 mice.

**Supplementary Movie 7:** **2P-IVM of the brain of CX3CR1-GFP; Aqp4-mRuby3 mice differentiates GFP^+^ border associated macrophages (BAMs) and microglia based on their position to the glia limitans.**

Representative 3D reconstruction of the brain surface from a CX3CR1-GFP; Aqp4-mRuby3 reporter mouse imaged by 2P-IVM though a cranial window preparation. The video shows first a top overview (XY MIP) of the cranial window. Due to the defined acquisition settings with excitation laser wavelength of 1000 nm, the SHG from the dura mater collagen type I fibers is faintly visible in green. After rotation, a lateral view (YZ MIP) is shown where CX3CR1-GFP^+^ macrophages (green) are found on top of the AQP4-mRuby3^+^ glia limitans (red), whereas CX3CR1-GFP^+^ microglia (green) are visible in the bottom compartment corresponding to the brain parenchyma. Digitally removing the upper region of the stack and focusing on an optical section below the superficial glia limitans, we can observe the distribution of microglia (white circles) relative to glia limitans covered-penetrating vessels. In the left region of the section, a microglial cell body wrapping a penetrating blood vessel is visible. This is the source video from which Fig. 7c-e were taken. Data are representative of 2 mice.

**Supplementary Movie 8: 2P-IVM of CX3CR1-GFP; Aqp4-mRuby3 mice allows *in vivo* visualization of the dynamic contacts of the different GFP^+^ myeloid cells with the glia limitans.**

The first movie is a 60-minute video from a cranial window showing an optical section located below the superficial glia limitans (red), where microglia (white circles) can be observed embedded in the parenchyma and perivascular BAMs are located within the perivascular areas (pink circles), facing the perivascular glia limitans. Some round CX3CR1-GFP^+^ cells, most likely peripheral monocytes (green), can be visualized rapidly circulating within blood vessel lumens. This is the source video from which Fig. 7d was taken. The second 40-minute video shows a deeper optical section depicting the dynamic spatial interaction between brain microglia and the glia limitans covered-blood vessels (white arrows). This is the source video from which Fig. 7e was taken. The third 40-minute video shows a z-plane located at the expected level of dura mater collagen I layers, visible by their SHG as green parallel fibers with faint signal due to the settings used (λ_ex_=1000 nm). BAMs are resting on the SHG (white circles). This is the source video from which Fig. 7c was taken. The fourth 40-minute video shows an optical section focusing on the superficial glia limitans of the spinal cord. Three BAMs (white arrows) are observed that remain largely immobile. This is the source video from which Fig. 7h was taken. MO = macrophage. Data are representative of 2 mice for each surgical preparation.

**Supplementary Movie 9: 2P-IVM of the spinal cord of CX3CR1-GFP; Aqp4-mRuby3 mice differentiates GFP^+^ border associated macrophages (BAMs) and microglia based on their position to the glia limitans.** Representative 3D reconstruction of the cervical spinal cord surface from a CX3CR1-GFP; Aqp4-mRuby3 reporter mouse imaged with 2P-IVM spinal cord window. The video first shows a top overview (XY MIP) of the spinal cord window. After rotation, a YZ MIP (lateral view) allows to visualize the AQP4-mRuby3^+^ glia limitans in red separating two compartments. The top compartment is the large subarachnoid space (SAS), where we can observe the shape of the dorsal vein lumen (DV) delineated by the CX3CR1^+^ BAMs in green, resting on top of this vein. The bottom compartment corresponds to the spinal cord parenchyma where microglial cells are located (green). Subsequently, the video displays in closer detail a ROI (XY MIP) including a group of three BAMs on the surface of the spinal cord, lateral to the DV. This is the source video from which Fig. 7h-j were taken. DV = dorsal vein. Data are representative of 2 mice.

**Supplementary Movie 10: 2P-IVM of GFP^+^** **2D2 CD4 T cell dynamics above and below the glia limitans in the spinal cord during EAE in CNS border reporter mice.**

Representative 2P-IVM video of the cervical spinal cord of a female border reporter mouse at the peak of EAE (score 1). Female CNS border reporter mice were previously transferred with 2 x 10^5^ naïve GFP^+^ 2D2 CD4 T cells, injected intravenously at day -1. XY MIP after removal of SHG (left panel) and YZ MIP (right panel) image sequences showing the dynamics of GFP^+^ 2D2 CD4 T cells (green) crawling above or below the AQP4-mRuby3 signal indicating the glia limitans visible in red (GL). The VE-cadherin-GFP signal (green) shows the adherens junctions from the endothelial cells and meningeal fibroblasts. The SHG from the subpial, dura mater and trabeculae collagen type I bundles is visible in blue. This is the source video from which Fig. 9b-e was taken. BV = blood vessel, SAS = subarachnoid space. Data are representative of 3 mice.

**Supplementary Movie 11: 2P-IVM of a 2D2 Th1 cell extravasating from a pial vessel during EAE.**

Representative 2P-IVM image series of the cervical spinal cord of a female border reporter mouse at the peak of EAE (18-19 days p.i.) is shown. During 2P-IVM, 5x10^6^ Deep-Red Cell Tracker labeled 2D2 Th1 cells were injected via a carotid artery catheter. XY MIP imaging sequence showing a 2D2 Th1 cell (white) extravasating from pial vessel into the SAS. The AQP4-mRuby3 signal (red) shows the glia limitans. The VE-cadherin-GFP signal (green) shows the adherens junctions from the endothelial cells and meningeal fibroblasts. SHG from the subpial collagen type I bundles is visible in blue. Light blue arrow points to the extravasating 2D2 Th1 cell. SAS = subarachnoid space; BV = blood vessel. Data are representative of 3 mice.

**Supplementary Movie 12: Dynamics of CD4 T cells crawling in enlarged perivascular spaces of cervical spinal cord venules during EAE in CNS border reporter mice.**

Representative 2P-IVM video of the cervical spinal cord of a female border reporter mouse at the peak of EAE (score 2). Mice received an intravenous injection of 5 x 10^5^ naïve GFP^+^ 2D2 CD4 T cells one day prior to inducing aEAE. The sequence shows first a XY MIP of the imaging window. Due to the acquisition with simultaneous excitation laser wavelengths of 1045 nm and 920 nm the SHG from the dura mater collagen type I fibers is visible in blue and green on the surface. The VE-cadherin-GFP signal from adherens junctions of the endothelial cells and the arachnoid and pia mater fibroblasts and the numerous 2D2 CD4 T cells are observed in green. AQP4-mRuby3 is visible in red at the expected level of the glia limitans. A complete rendering of the z-stack from top to bottom is shown using an optical slicer. XY and YZ MIPs focus on the spinal cord venule and the glia limitans, where an enlarged perivascular space is visible, identified between the endothelial cells and the glia limitans. GFP⁺ 2D2 CD4⁺ T cells accumulate in this space and are crawling within this confined region. Data are representative of 2 mice. BV = blood vessel, GL = glia limitans.

**Supplementary Movie 13: Dynamics of OT-I cells in the spinal cord during CNS immune surveillance.**

Naïve GFP^+^ OT-I cells were adoptively transferred into female border reporter mice followed 24 hours later by a peripheral challenge with LCMV-OVA. Representative 2P-IVM image series of the cervical spinal cord of a border reporter mouse on day 7 after LCMV-OVA infection is shown. XY MIP (left panel) and YZ MIP (right panel) image sequences showing the dynamic behavior of OT-I cells (green) during CNS immune surveillance. The AQP4-mRuby3 signal from the glia limitans is shown in red. Blue circle highlights an OT-I cell crawling on top of the glia limitans within the CNS border compartment (SAS or subpial space). SAS = subarachnoid space. Data are representative of 3 mice.

**Supplementary Movie 14: Dynamics of OT-I cells in the spinal cord during neuroinflammation.**

Naïve GFP^+^ OT-I cells were adoptively transferred into female border reporter; ODC-OVA mice followed 24 hours later by a peripheral challenge with LCMV-OVA. Representative 2P-IVM image series of the cervical spinal cord on day 7 after LCMV-OVA infection, at the peak of clinical neuroinflammation is shown. XY MIP (left panel) and YZ MIP (right panel) image sequences showing the dynamic behavior of OT-I cells (green) during neuroinflammation. The AQP4-mRuby3 signal from the glia limitans is shown in red. Blue circle highlights an OT-I cell probing on top of the glia limitans within the CNS border compartment (SAS or subpial space). Pink circle highlights an OT-I cell below the glia limitans within the spinal cord parenchyma. Data are representative of 3 mice.

**Supplementary Movie 15: 2P-IVM of an OT-I cell crossing the glia limitans during neuroinflammation.**

Representative image sequence of 2P-IVM of the cervical spinal cord of a female border reporter; ODC-OVA mouse at peak (7 days after LCMV-OVA infection) of OT-I cell mediated autoimmune neuroinflammation is shown. XY MIP (left panel) and YZ MIP (right panel) image sequences showing OT-I cells (green) on top and below the AQP4-mRuby3^+^ glia limitans (red). Blue circle highlights one OT-I cell crossing the glia limitans from the CNS border compartment (SAS or subpial space) into the CNS parenchyma. Data are representative of three independent experiments.
